# Matrix Metalloproteinase-2 Knockout and Heterozygote Mice Are Protected from Hydronephrosis and Kidney Fibrosis after Unilateral Ureteral Obstruction

**DOI:** 10.1371/journal.pone.0143390

**Published:** 2015-12-16

**Authors:** Maria K. Tveitarås, Trude Skogstrand, Sabine Leh, Frank Helle, Bjarne M. Iversen, Christos Chatziantoniou, Rolf K. Reed, Michael Hultström

**Affiliations:** 1 Department of Biomedicine, University of Bergen, Bergen, Norway; 2 Department of Clinical Medicine, University of Bergen, Bergen, Norway; 3 Department of Medicine, Haukeland University Hospital, Bergen, Norway; 4 Department of Pathology, Haukeland University Hospital, Bergen, Norway; 5 Inserm UMR 702, Université Pierre et Marie Curie, Paris VI, Paris, France; 6 Center for Cancer Biomarkers, CCBIO, University of Bergen, Bergen, Norway; 7 Department of Medical Cellbiology, Uppsala University, Uppsala, Sweden; 8 Anaesthesiology and Intensive Care Medicine, Department of Surgical Sciences, Uppsala University, Uppsala, Sweden; University of Patras, GREECE

## Abstract

Matrix Metalloproteinase-2 (Mmp2) is a collagenase known to be important in the development of renal fibrosis. In unilateral ureteral obstruction (UUO) the obstructed kidney (OK) develops fibrosis, while the contralateral (CL) does not. In this study we investigated the effect of UUO on gene expression, fibrosis and pelvic remodeling in the kidneys of Mmp2 deficient mice (Mmp2-/-), heterozygous animals (Mmp2+/-) and wild-type mice (Mmp2+/+). Sham operated animals served as controls (Cntrl). UUO was prepared under isoflurane anaesthesia, and the animals were sacrificed after one week. UUO caused hydronephrosis, dilation of renal tubules, loss of parenchymal thickness, and fibrosis. Damage was most severe in Mmp2+/+ mice, while both Mmp2-/- and Mmp2+/- groups showed considerably milder hydronephrosis, no tubular necrosis, and less tubular dilation. Picrosirius red quantification of fibrous collagen showed 1.63±0.25% positivity in OK and 0.29±0.11% in CL (p<0.05) of Mmp2+/+, Mmp2-/- OK and Mmp2-/- CL exhibited only 0.49±0.09% and 0.23±0.04% (p<0.05) positivity, respectively. Mmp2+/- OK and Mmp2+/- CL showed 0.43±0.09% and 0.22±0.06% (p<0.05) positivity, respectively. Transcriptomic analysis showed that 26 genes (out of 48 examined) were differentially expressed by ANOVA (p<0.05). 25 genes were upregulated in Mmp2+/+ OK compared to Mmp2+/+ CL: Adamts1, -2, Col1a1, -2, -3a1, -4a1, -5a1, -5a2, Dcn, Fbln1, -5, Fmod, Fn1, Itga2, Loxl1, Mgp, Mmp2, -3, Nid1, Pdgfb, Spp1, Tgfb1, Timp2, Trf, Vim. In Mmp2-/- and Mmp2+/- 18 and 12 genes were expressed differentially between OK and CL, respectively. Only Mmp2 was differentially regulated when comparing Mmp2-/- OK and Mmp2+/- OK. Under stress, it appears that Mmp2+/- OK responds with less Mmp2 upregulation than Mmp2+/+ OK, suggesting that there is a threshold level of Mmp2 necessary for damage and fibrosis to occur. In conclusion, reduced Mmp2 expression during UUO protects mice against hydronephrosis and renal fibrosis.

## Introduction

Obstructive nephropathy is a common cause of kidney damage and renal insufficiency, both in congenital obstructive nephropathy in children [1], and acquired obstruction caused by kidney stones, malignancies and benign prostate hyperplasia [2]. In rodents unilateral ureteral obstruction (UUO) is a well-studied model that leads to hydronephrosis with tubular dilation, cortical atrophy and fibrosis. UUO is interesting both as a model of ureteral obstruction, and for studying the fibrotic process as such [3]. The development and degree of fibrosis is considered to be one of the most reliable prognostic markers for loss of kidney function and progression towards end stage renal disease (ESRD) [4].

Matrix Metalloproteinase-2 (Mmp2), also known as gelatinase-A, is a 72 kDa collagenase that is important in extracellular matrix metabolism. Mmp2 cleaves type IV collagen, and degrades already denatured collagens [5]. In the kidney, Mmp2 is upregulated in several pathological states [6–9]. Inhibition of Mmp2 activity results in disparate outcomes depending on the phase of kidney disease studied and on the underlying cause [10,11]. For example, it has been shown that Mmp2 facilitates fibrosis by participating in epithelial to mesenchymal transition [12]. Mmp2 has also been found to be involved in tubular repair after acute kidney injury (AKI) [13], and Mmp2 deficiency protects against ischemia-reperfusion AKI [14].

Mmp2 knockout mice (Mmp2-/-) do not show major anatomical abnormalities, but are born smaller and grow more slowly than the wild type (Mmp2+/+), suggesting that Mmp2 is important for fetal development and growth [15]. Knockout of the Mmp2 gene occurs in exon 1, resulting in no Mmp2 expression neither at the RNA nor the protein level [15]. The Mmp2-/- mice show reduced angiogenic response in oxygen-induced retinopathy [16], and are more susceptible to diabetic nephropathy [17]. However, they are protected against haemorrhagic transformation during the early stages of cerebral ischemia and reperfusion [18].

Since UUO damage is closely connected to the remodeling of the renal pelvis and the deformation of the kidney parenchyma, we hypothesized that Mmp2-deficiency would protect the obstructed kidney (OK). However, a recent study of pharmacological inhibition in the UUO model showed increased fibrosis, while cellular infiltration was decreased [19]. The aim of the present study was to investigate the effect of homozygous and heterozygous genetic inactivation of Mmp2 on gene expression, fibrosis and pelvic remodeling in the kidneys of mice after one week of UUO.

The fibrotic process was investigated in knockout animals (Mmp2-/-), heterozygotes (Mmp2+/-) and wild-type C57Black6J (Mmp2+/+) mice. In addition, sham operated individuals from each group served as controls (Cntrl-/-, Cntrl+/-, Cntrl+/+). The genes selected for investigation in this study were chosen due to their involvement in fibrosis and renal damage.

## Materials and Methods

### Animals

Mmp2 deficient C57BL/6J mice were generously provided by Dr. Werb [15] at Inserm UMR 702, Université Pierre et Marie Curie, Paris, and later transferred to Bergen for use in the present project. The animals were kept and bred at the animal facility at the Department of Biomedicine in Bergen. The study consisted of 6 groups; Mmp2+/+ Control (Cntrl+/+) (n = 10) and UUO (n = 10), Mmp2+/- Control (Cntrl+/-) (n = 10) and UUO (n = 11), and Mmp2-/- Control (Cntrl-/-) (n = 10) and UUO (n = 9) (Table 1). The control groups were sham operated.

**Table 1:** Bodyweight and age of the mice at the start of the experiment.

	Mmp2+/+		Mmp2+/-		Mmp2-/-	
	Control	UUO	Control	UUO	Control	UUO
**n =**	10	10	10	11	10	9
**Bodyweight (g)**	31±0.4	23±0.3	35±1	38±2	29±2	29±2
**Age (weeks)**	14±0	9±0	19±1	37±2	20±2	41±6

### Ethics Statement

The experiments were conducted in accordance with the guidelines of, and with approval obtained from the Norwegian State Board for Biological Experiments with Living Animals (Approval No: 2009–1899). All surgery was carried out under isoflurane anaesthesia.

### Ureteral obstruction

UUO was prepared under isoflurane anaesthesia. The left ureter was identified through a subcostal incision, and obstructed using a silk ligature at the level of the lower pole of the kidney. The animals were sacrificed under isoflurane anaesthesia one week after obstruction. The abdominal aorta was dissected and cannulated in order to perfuse the animal with ice-cold PBS before the kidneys were removed. The kidneys were cut in transverse slices that were either stabilised in RNA-later, or fixed in 4% formaldehyde, processed and embedded in paraffin.

### Histology

Morphological damage was investigated by light microscopy using 3 μm sections, stained with Periodic Acid-Schiff (PAS). In order to monitor the amount of collagen in the different experimental groups, 7 μm sections were stained with Picrosirius Red and examined according to our previously published protocol [9]. Briefly, digital images were captured randomly under constant polarized light in a Leica DMLB microscope connected to a CCD ColorView IIIu camera. Image acquisition and analysis was performed using CellD version 2.4. Intensity was separated as a gray-scale image from the HSI colour-space. The detection threshold was the same for all images. Collagen content was expressed as percent positive pixels of total pixels.

### Quantitative RTPCR

RNA was extracted from the kidneys using an RNeasy mini kit (Qiagen, West Sussex, UK) as described in the protocol provided by the manufacturer, and cDNA was synthesised from RNA using Reverse Transcriptase Core Kit obtained from Eurogentec (Seraing, Belgium). A custom-made Low Density Array (LDA) from Applied Biosystems was used to determine the mRNA expression levels of a selection of genes. The method is based on the well-established quantitative RT-PCR (QRT-PCR) technique, however, LDA has the benefit of enabling quantification of several genes simultaneously and at the same time maintaining the sensitivity of QRT-PCR [20]. 18s ribosomal RNA was used as a standard. In addition Gapdh, Tbp, Pgk1 and Ppia were used as housekeeping genes.

### Statistical analysis

Data is presented as means ± standard error of the mean (SEM), except for comparison of the expression of individual genes where the fold-change is used. The probability of chance difference was tested using ANOVA, with Fisher’s test and à priori contrasts to test individual comparisons. Comparisons between CL and OK were paired. The Bonferroni correction was used for gene-expression data, separately across samples and across genes for each comparison made. P < 0.05 was accepted as statistically significant.

## Results

### Kidney damage in UUO

**Fig 1:**
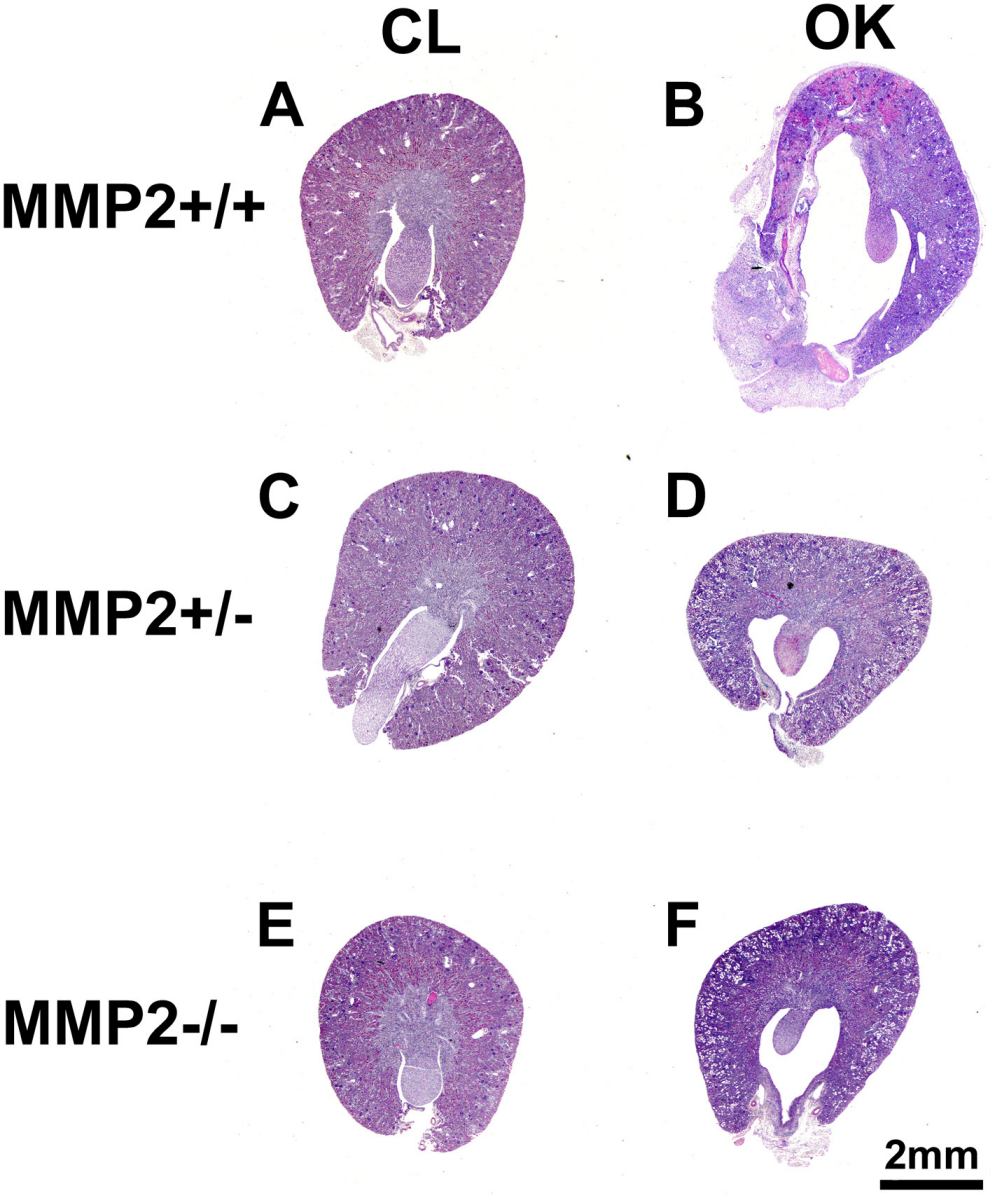
Representative images of Period Acid-Schiff (PAS) stained transversal sections showing increased morphological damage in the Mmp2+/+ obstructed kidney (OK, 1B) compared to the other groups (1 D, F). The Mmp2+/+ OK (1B) shows severe hydronephrosis and inflammation of the renal pelvis, while the Mmp2-/- OK (1F) and Mmp2+/- OK (1D) only display slight dilatation and minimal inflammation. The contralateral kidneys (CL, 1 A, C, E) do not differ from each other and show normal morphology.

**Fig 2:**
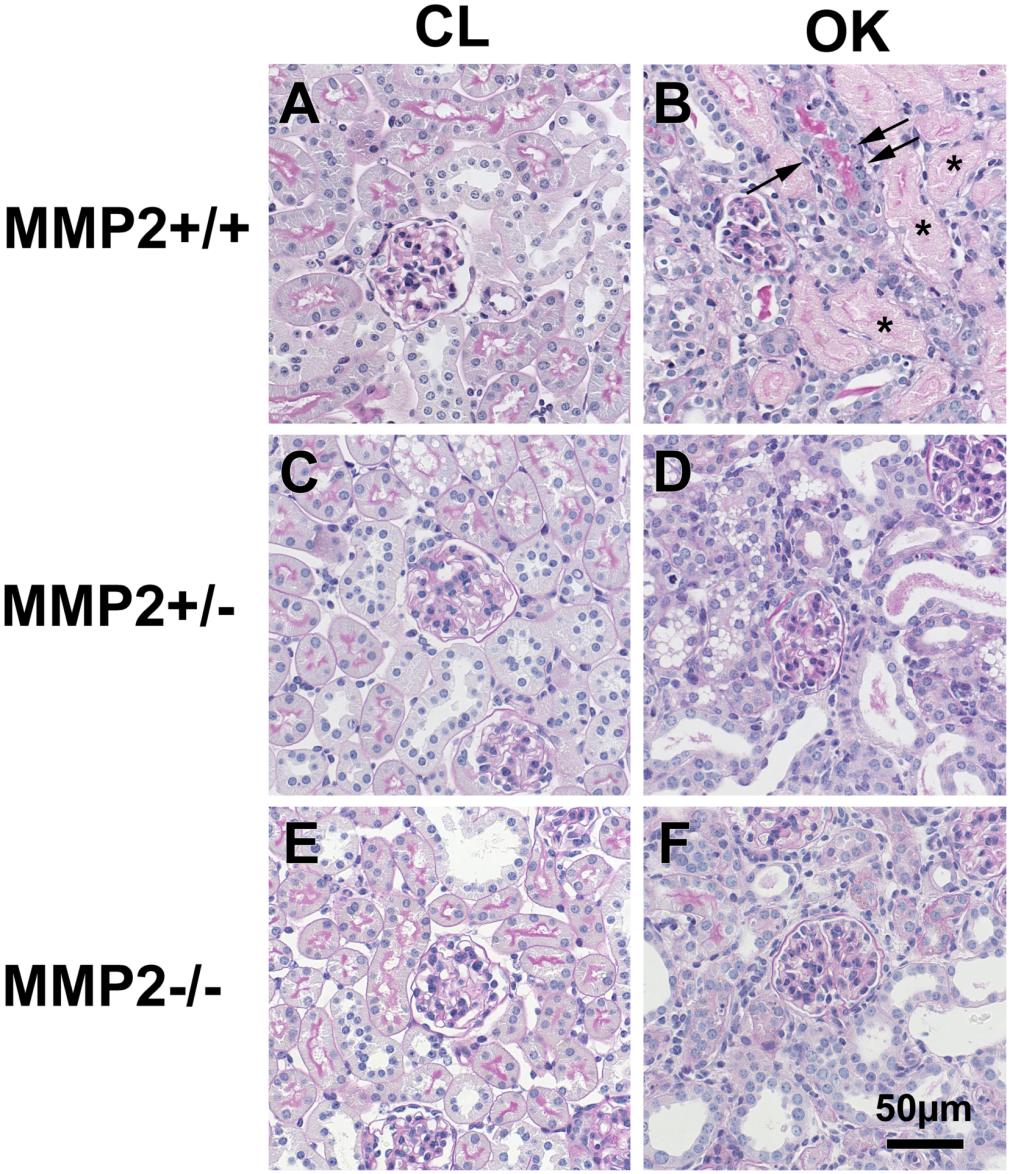
Morphological damage in the obstructed kidney (OK, 2B, D, F) compared to the contralateral kidney (CL, 2 A, C, E). All OK kidneys show tubular dilatation, flattened tubular epithelium with loss of brush border and reactive nuclear enlargement. Only Mmp2+/+ OK (2B) shows necrotic tubules (asterix) and many apoptotic cells (arrows). The morphology of the CL kidney is normal. (PAS stain).

**Fig 3:**
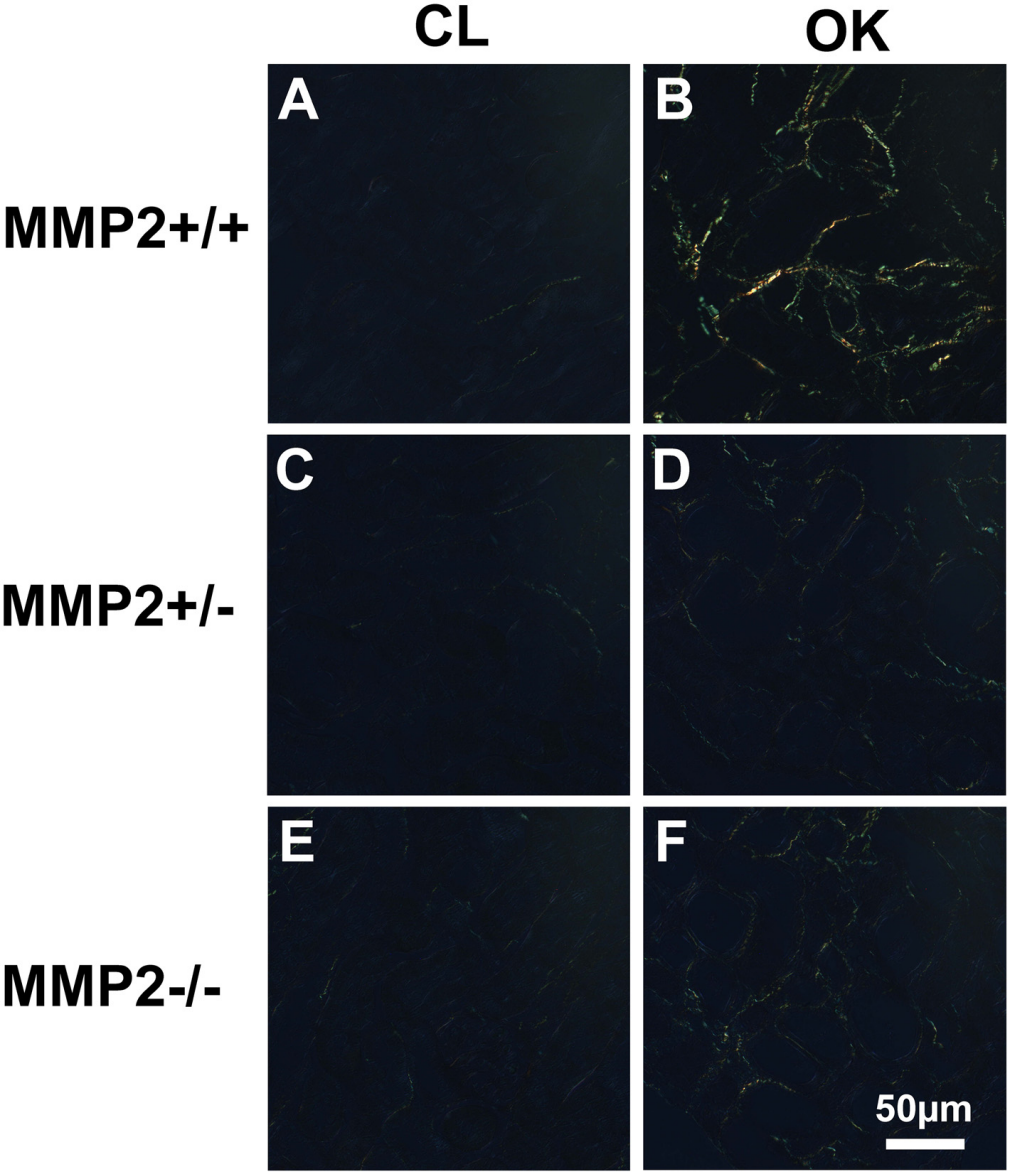
Representative images of Picrosirius Red stained sections under polarised light from the obstructed kidney (OK, 3 B, D, F) compared to the contralateral kidney (CL, 3 A, C, E). There is a higher level of collagen in Mmp2+/+ OK (3 B) compared to OK in both Mmp2+/- and Mmp2-/- (3 D, F). Only minimal levels of collagen are detected in CL kidneys across all groups (3 A, C, E).

**Fig 4:**
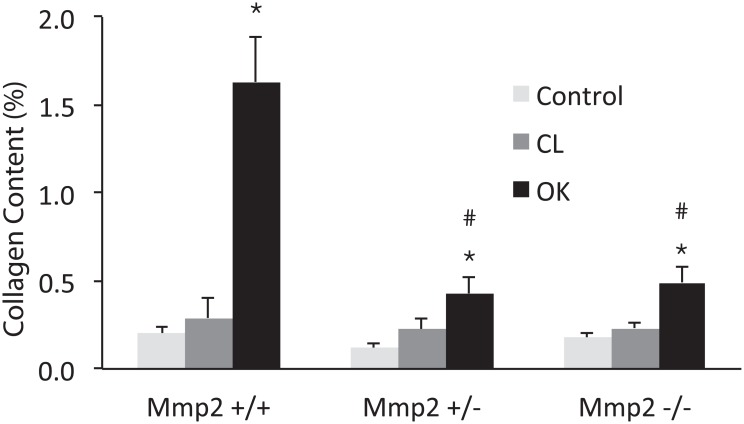
Image analysis of collagen in Picrosirius Red stained sections under polarised light. Collagen content is expressed as the percent of positive pixels to all pixels. * denotes P<0.05 compared to the contralateral kidney (CL). # denotes P<0.05 compared to OK in Mmp2+/+.

A total of 60 mice were used in the UUO experiment (Table 1). UUO caused severe hydronephrosis in Mmp2+/+, which was accompanied by tubular dilation, necrosis and atrophy, while Mmp2-/- and Mmp2+/- showed considerably milder hydronephrosis, no tubular necrosis, and less tubular dilation (Figs 1 and 2). Automatic image analysis of collagen content in Picrosirius Red stained sections showed 1.63±0.25% positivity in OK and 0.29±0.11% in CL (p<<0.05) of Mmp2+/+, whereas Mmp2-/- OK and Mmp2-/- CL only showed 0.49±0.09% and 0.23±0.04% (p<0.05) positivity, respectively, and Mmp2+/- OK and Mmp2+/- CL exhibited 0.43±0.09% and 0.22±0.06% (p<0.05) positivity, respectively (Figs 3 and 4). There were no significant discrepancies between the levels of fibrosis in the control kidneys and the CL kidneys in any of the groups.

### Gene expression

Using RTPCR, 26 out of 48 genes examined showed differential expression when ANOVA was applied across groups (Table 2). None of the housekeeping genes showed significant changes, and the results were comparable when using the different genes for normalisation. Data are presented using 18S as standard. There was no difference in gene expression between CL and control kidneys in any of the groups.

**Table 2:** Differential gene expression between strains (Mmp2 +/+, +/-, and -/-), and as paired comparisons between the obstructed (OK) and contralateral kidneys (CL) of the same animal.

Gene	Average	OK-/-	OK-/-	OK+/-	OK-/-	CL-/-	OK+/-	CL+/-	OK+/+	CL+/+	Cntrl-/-	Cntrl-/-	Cntrl+/-
	CT-value	vs	vs	vs	vs	vs	vs	vs	vs	vs	vs	vs	vs
	± SEM	OK+/+	OK+/-	OK+/+	CL-/-	Cntrl-/-	CL+/-	Cntrl+/-	CL+/+	Cntrl+/+	Cntrl+/+	Cntrl+/-	Cntrl+/+
Mmp2 †	33.05 ± 0.28	-7.060 *	-5.774 *	-1.287 *	-0.907	0.704	3.973 *	-0.345	4.079 *	0.455	-2.323 *	-1.942 *	-0.381
Adamts2 †	31.06 ± 0.22	-0.666	0.479	-1.146 *	2.748 *	0.532	3.122 *	0.247	3.867 *	0.033	-0.046	0.569	-0.615
Dcn †	28.22 ± 0.18	-0.533	0.534	-1.067 *	1.774 *	0.914	1.269	0.801	3.078 *	-0.567	-0.710	-0.084	-0.626
Mgp †	24.65 ± 0.32	0.829	0.421	0.408	2.853 *	1.599	3.673 *	0.420	3.555 *	-0.152	-0.219	0.062	-0.280
Adamts1 †	29.84 ± 0.24	0.726	0.435	0.291	3.840 *	-0.241	3.591 *	-0.626	2.463 *	-0.012	-0.423	-0.199	-0.224
Spp1 †	23.72 ± 0.28	0.870	0.483	0.387	3.409 *	1.432	4.996 *	-0.538	3.337 *	0.887	0.255	0.100	0.154
Col4a1 †	25.82 ± 0.16	-0.187	0.340	-0.527	2.463 *	0.071	0.977	1.208	2.160 *	0.349	-0.212	-0.009	-0.203
Fn1 †	29.61 ± 0.25	-0.782	0.149	-0.930	1.713	1.742	4.074 *	-0.643	4.371 *	0.295	0.429	0.125	0.304
Col1a2 †	26.48 ± 0.23	-0.845	0.475	-1.320	3.921 *	-0.011	3.598 *	-0.133	4.415 *	0.123	-0.217	0.029	-0.246
Vim †	27.38 ± 0.17	-0.369	0.403	-0.772	2.109 *	0.633	1.032	1.342	3.098 *	0.065	0.051	0.034	0.016
Nid1 †	29.28 ± 0.15	-0.358	0.132	-0.490	1.578 *	0.620	1.296 *	0.774	1.898 *	0.764	0.106	0.004	0.103
Mmp3 †	32.99 ± 0.29	0.052	0.600	-0.548	4.706 *	0.870	4.741 *	-0.138	4.228 *	0.029	-1.267	-0.374	-0.893
Trf †	30.29 ± 0.23	0.461	0.530	-0.069	1.550 *	0.221	1.201	0.656	2.591 *	0.025	1.305	0.616	0.689
Col3a1 †	28.04 ± 0.31	-0.610	0.958	-1.568	5.332 *	0.239	4.743 *	-0.003	5.177 *	0.561	-0.444	0.126	-0.570
Col1a1 †	29.56 ± 0.21	-0.953	0.671	-1.624	4.894 *	0.349	4.655 *	-0.306	4.914 *	0.534	-0.747	-0.223	-0.524
Loxl1 †	29.77 ± 0.18	-0.088	0.172	-0.261	1.518	1.255	1.147	1.518	2.978 *	-0.067	0.049	0.064	-0.014
Tgfb1 †	28.97 ± 0.16	0.316	0.329	-0.014	2.336 *	0.309	0.983	1.514	2.450 *	-0.003	0.118	0.181	-0.063
Col5a1 †	32.47 ± 0.23	-1.296	0.050	-1.346	1.573	1.698	2.074 *	1.065	3.823 *	1.038	0.293	-0.082	0.376
Pdgfb †	29.29 ± 0.22	0.640	0.237	0.404	1.701 *	0.897	1.189	1.077	1.682 *	0.191	-0.085	-0.094	0.010
Fbln5 †	28.53 ± 0.14	-0.462	-0.029	-0.433	0.824	0.238	0.905	-0.134	1.575 *	0.136	0.186	-0.321	0.507
Fmod †	31.42 ± 0.10	-1.316 *	-0.260	-1.057 *	0.309	0.057	0.768	-0.384	1.217 *	-0.284	-0.750	-0.242	-0.508
Mmp9 †	34.56 ± 0.24	0.568	0.864	-0.296	2.160 *	1.186	0.967	2.494	5.068	-2.000	0.289	0.980	-0.690
Fbln1 †	30.71 ± 0.17	0.374	0.763	-0.389	1.666	1.588	1.223	1.119	2.411 *	-0.161	-0.629	-0.148	-0.481
Timp2 †	29.50 ± 0.13	0.319	0.488	-0.169	1.455 *	0.797	0.952	0.885	1.424 *	0.308	-0.201	0.072	-0.274
Col5a2 †	30.88 ± 0.18	-0.571	0.346	-0.917	1.280	1.092	0.856	1.236	2.922 *	-0.026	-0.048	0.065	-0.114
Itga2 †	34.10 ± 0.25	-0.603	0.568	-1.171	3.203 *	0.285	2.148	-0.567	3.516 *	-0.231	-0.807	-1.340	0.532
Lgals3	27.31 ± 0.20	-0.079	0.289	-0.368	1.423	0.684	0.699	1.264	2.212	-0.271	-0.245	0.144	-0.389
Timp1	31.18 ± 0.42	-0.642	0.328	-0.970	3.155	4.151	3.867	3.489	7.435	-0.743	-1.256	0.378	-1.634
Itgb3	33.51 ± 0.22	-0.273	0.262	-0.534	1.860	0.322	0.406	1.516	2.377	0.911	0.833	0.002	0.831
Pdgfa	31.10 ± 0.14	0.383	0.366	0.017	0.975	0.782	0.815	0.511	1.418	-0.204	-0.159	-0.064	-0.095
Il1b	32.40 ± 0.19	-0.147	0.259	-0.406	1.526	1.701	1.332	1.269	3.335	-0.494	-0.533	-0.368	-0.166
Pgk1	26.20 ± 0.12	0.040	-0.164	0.204	-0.438	-0.175	0.007	-0.611	-0.886	-0.002	-0.235	-0.155	-0.080
Itgav	27.80 ± 0.19	0.081	-0.048	0.129	0.953	0.236	0.942	0.410	1.513	-0.128	0.277	0.115	0.162
Vegfa	27.77 ± 0.08	-0.238	-0.073	-0.165	-0.459	-0.148	-0.196	-0.469	-0.769	0.295	-0.104	-0.131	0.027
Gapdh	22.53 ± 0.21	-0.034	-0.031	-0.003	-0.162	-0.205	0.033	-0.434	-0.439	-0.202	-0.308	-0.065	-0.243
Vegfc	32.97 ± 0.11	0.159	0.458	-0.299	0.649	0.427	0.557	-0.275	0.766	-0.072	-0.224	-0.337	0.113
Nkap	30.48 ± 0.09	0.329	0.132	0.197	0.557	0.061	0.628	-0.052	0.247	-0.090	-0.132	0.089	-0.221
Itga10	34.71 ± 0.12	0.009	0.341	-0.331	0.506	-0.373	0.308	-0.190	1.233	-0.230	0.880	0.326	0.554
Vegfb	29.01 ± 0.12	0.489	0.056	0.434	-0.465	0.116	-0.082	-0.249	-0.814	0.074	0.098	0.074	0.024
Itga11	34.60 ± 0.16	-0.295	0.767	-1.062	1.445	0.832	1.340	0.034	2.641	-0.138	-0.069	-0.136	0.067
Pdgfc	30.78 ± 0.11	0.203	0.140	0.063	-0.036	-0.123	0.309	-0.418	-0.412	0.191	0.140	0.190	-0.049
Tbp	30.20 ± 0.70	0.161	0.001	0.160	0.322	0.089	0.375	-0.218	0.144	-0.070	-0.175	-0.252	0.077
Ppia	23.73 ± 0.10	0.214	0.221	-0.007	0.294	0.060	0.217	-0.175	0.014	-0.017	-0.142	-0.090	-0.052
Itgb1bp1	29.15 ± 0.06	0.272	0.086	0.186	0.322	-0.196	0.161	-0.029	-0.197	0.001	-0.051	0.092	-0.142
Tff3	35.70 ± 0.14	NA	NA	NA	NA	NA	NA	NA	0.253	0.679	NA	NA	1.029
Il1a	35.93 ± 0.31	-0.429	1.094	-1.523	1.657	-1.016	-0.533	0.624	1.242	-0.540	-0.368	0.544	-0.912
Matn1	33.92 ± 1.25	NA	NA	NA	NA	NA	NA	NA	NA	NA	NA	NA	NA

Cntrl denotes kidneys from control animals without obstruction.

^†^ denotes significant variations between all groups, P<0.05 by ANOVA.

* denotes P<0.05 for the individual comparison.

The greatest difference in gene expressions was seen when comparing the OK and CL in the Mmp2+/+ mice, where 25 genes were significantly altered (Table 2). A comparison of Mmp2-/- OK vs Mmp2-/- CL identified 18 significantly altered genes. Mmp2+/- OK displayed 12 significantly altered genes when compared to Mmp2+/- CL. Cntrl+/- did not differ from Cntrl+/+ in any of the 48 tested genes. After UUO, Mmp2 expression was 7-fold and 5.5-fold induced in Mmp2+/+ OK and Mmp2+/- OK, respectively, when compared to Mmp2-/- OK. Comparisons between Mmp2-/- OK vs Mmp2-/- CL, and Mmp2+/- OK vs Mmp2+/- CL showed that five genes in common did not respond in the same manner as in the Mmp2+/+ OK vs Mmp2+/+ CL comparison. These genes are Loxl1, Fbln5, Fmod, Fbln1 and Col5a2. None of these genes were significantly altered after UUO in Mmp2 -/- and Mmp2+/- mice. In contrast, all five genes were upregulated in Mmp2+/+ mice. Fmod is downregulated in both Mmp2-/- and Mmp2+/- OK groups compared to Mmp2+/+ OK. Mmp9 showed a 2.2-fold upregulation in Mmp2-/- OK when compared to Mmp2-/- CL mice. Only Mmp2 was differentially regulated when comparing Mmp2-/- OK and Mmp2+/- OK. In addition to the housekeeping genes, 17 genes were not differentially expressed between any of the groups.

## Discussion

The present study demonstrates that Mmp2-/- and Mmp2+/- mice display less pelvic-remodelling and fibrosis compared to Mmp2+/+ mice after UUO. Both Mmp2-/- and Mmp2+/- mice are protected, which may indicate an important effect of gene-dose. Furthermore, Mmp2+/+ mice displayed more histological damage and showed the highest response in gene expression following UUO, with 25 genes significantly altered when comparing OK to CL. Both Mmp2-/- and Mmp2+/- animals gave a milder response to UUO, showing 18 and 12 significantly altered genes after UUO, respectively. The main findings in this study are similar to those of Du et al., described in a comprehensive paper in which they also reported reduced infiltration of immune cells, and improved immunohistochemistry for a number of collagens and renal injury markers [21]. Our RT-PCR results correlate to those of Du et al with similar expression patterns for Mmp2, Mmp9, Timp1 and Timp2. However, our study presents a wider range of molecular markers, and also data from heterozygotes beyond what has been previously reported.

When comparing the control kidneys between the groups, the lack of Mmp2 does not seem to affect the amount of collagen when the kidney is not under stress (Fig 4). As expected, the level of Mmp2 in both Cntrl+/- and Cntrl+/+ animals is higher relative to the Cntrl-/- group. There is no significant difference in Mmp2 expression between wild type and heterozygotes under control conditions. Mmp2 gene expression in Mmp2+/- mice is not upregulated in OK as much as in the Mmp2+/+ OK. This could suggest that there is a threshold level of Mmp2 necessary for damage and fibrosis to occur and this level might not be reached in the Mmp2+/- mice, thus resulting in protection of the kidneys in Mmp2+/- mice as well. Taken together with the fact that Mmp2 upregulation is required for regulation of several of the genes studied here, this could point towards a cascade effect where a threshold level of Mmp2 is important for initiation of fibrogenesis.

Some of the genes utilised in our study have been investigated previously in similar settings, and could help elucidate the role of Mmp2 after UUO. The effect of pharmacological inhibition of Mmp2 seems to be dependent on the time of administration in UUO-induced renal fibrosis. Late administration of an inhibitor of Mmp2, TISAM, decreased the level of macrophage infiltration, while early administration did not, and contrary to our findings both early and late inhibition of Mmp2 resulted in accelerated fibrosis in the mouse kidney [19]. Inhibition by He4, an Mmp2/Mmp9 inhibitor and a pan-serine protease which has been shown to directly interact with and inhibit Mmp2 and Mmp9, improved the outcome after UUO [22]. He4-inhibition, and thus an increase in Mmp2 and Mmp9 mediated collagen I digestion, resulted in less fibrosis compared to the control group. Taken together, this suggests that genetic deficiency of Mmp2 affects the development of fibrosis by a different mechanism than pharmacological inhibition of Mmp2. This time dependency has also been described in a mouse model of Alport syndrome [10]. Early combination therapy with inhibitors of Mmp2, Mmp3 and Mmp9 significantly delayed the onset of proteinuria, while treatment after onset of proteinuria accelerated renal disease.

In addition, Mmp2 has been shown to affect vascular reactivity. For example endothelin-1 requires cleavage to produce the active hormone and Mmp2 contributes to this process [23]. Mmp2 has also been found to cleave adrenomedullin, resulting in production of a vasoconstrictor peptide [24], and this may be an underlying mechanism that leads to kidney damage [25]. One may suggest that Mmp2 deficiency decreases vasoconstriction and thus reduces kidney damage in UUO.

Mmp9 deficiency seems to have a positive effect during UUO [26], while pharmacological inhibition of Mmp9 has a time-dependent effect, with reduced epithelial to mesenchymal transition and fibrosis at the early or chronic stage, but not during the establishment of fibrosis [11]. The present data show upregulation of Mmp9 in the obstructed kidney in Mmp2-/- mice compared to CL, which may be a compensatory increase due to the lack of Mmp2 [10]. However, Mmp9 was not upregulated after UUO in Mmp2+/- animals, and since both strains showed less damage, it is unlikely that Mmp9 alone is responsible for the decreased fibrosis after UUO.

Decorin (Dcn) deficient mice have been reported to show greater damage than WT after UUO [27]. This could indicate that an upregulation of Dcn is a protective mechanism. However, while the expression of Dcn mRNA was increased after UUO in wild type and knockout mice, there was no effect in heterozygous animals. These results, therefore, do not allow one to draw firm conclusions regarding the potential protective role of Dcn. Timp2 is not differentially expressed between the groups, yet there is an upregulation of Timp2 in the OK kidney compared to the CL kidney in both Mmp2+/+ and Mmp2-/- mice, but not in the Mmp2+/- group. However, we do not have any basis to explain why Mmp2+/- mice regulate Timp2 differently under the stress of UUO. Earlier data showed that Timp2 promotes injury through activating Mmp2 [28]. In both Mmp2-/- and Mmp2+/- animals Timp2 has less Mmp2 to activate, which could be a mechanism behind the reduced rate of fibrosis and remodelling. Thus, even though Timp2 expression in OK and CL in Mmp2-/- and Mmp2+/- mice differ, the limited possibility of an increase in Mmp2 at the level of expression would probably reduce this effect. This may be the reason why they are also protected. In our study, Timp1 was not affected by UUO, and this is supported by evidence that elimination of Timp1 alone was not found to be sufficient in order to alter the severity of fibrosis during UUO [29].

The present results did not show consistent findings in the Pdgf system. This is not entirely surprising since the Pdgf system is important in kidney fibrosis [30] and in UUO [31,32], but it is not necessary for the development of fibrosis in the kidney [33]. There was no differential expression of Pdgfa or Pdgfc. On the other hand Pdgfb was differentially expressed after UUO in Mmp2-/- and Mmp2+/+ mice but not in Mmp2+/- animals. This is difficult to explain, especially since antagonists have a positive effect in several models of renal disease. Pdgfb, however, also plays a role in the repair of cellular damage [34].

Tff3, Vgfa, Vgfb, Vgfc, Il1b and Lgals3 were not differentially expressed in the present study even though they have been implicated in kidney disease. Specifically, Tff3 has been suggested as a diagnostic marker in CKD since it increases in both serum and urine during CKD progression [35]. Vegfs have been demonstrated to slow down the progression of renal injury in experimental models and can be administered in renoprotective therapy [36], but overstimulation can induce glomerular pathology. Il1b is known to stimulate proliferation of human renal fibroblasts as well as production of matrix proteins, while Lhals3 has been shown to protect against fibrosis [37,38].

The clinical treatment of ureteral obstruction is primarily surgical, but as a strategy for limiting the destruction of kidney tissue in progressive or recurring disease, some intervention against Mmp2 may be useful. It is further unlikely that reduced Mmp2 expression provides complete protection from the damage in UUO. There is no doubt that a longer period of UUO would lead to more severe damage, even in mice with decreased Mmp2 expression.

In conclusion, both homozygous and heterozygous genetic inactivation of Mmp2 protect mice against hydronephrosis and kidney fibrosis after UUO, as indicated by both histology and gene expression. The genetic mechanism seems to be a reduced ability to respond with Mmp2 upregulation under stress, and this may suggest that there is a threshold level of Mmp2 necessary for pelvic remodelling and genetic events leading to activation of fibrosis.
